# Supplementary material for: Hybrid Models and Biological Model Reduction with PyDSTool
Source: PLoS Comput Biol. 2012 Aug 9;8(8):e1002628. doi: 10.1371/journal.pcbi.1002628 (PMC3415397; doi:10.1371/journal.pcbi.1002628)
Supplement: Text S4 — Complete source code for the PyDSTool package (version 0.88.120504). Includes API documentation and help files linking to web pages. This file is identical to the current public release on Sourceforge.net. (ZIP) [file pcbi.1002628.s004.zip › PyDSTool/html/PyDSTool.Model.NonHybridModel-class.html]

xml version="1.0" encoding="ascii"?


PyDSTool.Model.NonHybridModel


| Home | Trees | Indices | Help | PyDSTool |
| --- | --- | --- | --- | --- |

Package PyDSTool ::
Module Model ::
Class NonHybridModel

[hide private]

[frames] | no frames]

# Class NonHybridModel

source code

```
object --+    
         |    
     Model --+
             |
            NonHybridModel
```

---


|  |  |
| --- | --- |
| Instance Methods | [hide private] |

|  |  |
| --- | --- |
| \_\_init\_\_(self, \*a, \*\*kw)  x.\_\_init\_\_(...) initializes x; see x.\_\_class\_\_.\_\_doc\_\_ for signature | source code |

|  |  |
| --- | --- |
| \_findTrajInitiator(self, end\_reasons, partition\_num, t0, xdict, gi=None, swRules=None) | source code |

|  |  |
| --- | --- |
| cleanupMemory(self)  Clean up memory usage from past runs of a solver that is interfaced through a dynamic link library. | source code |

|  |  |
| --- | --- |
| haveJacobian(self)  Returns True iff all objects in modelInfo have defined Jacobians. | source code |

|  |  |
| --- | --- |
| haveJacobian\_pars(self)  Returns True iff all objects in modelInfo have defined Jacobians. | source code |

|  |  |
| --- | --- |
| Rhs(self, t, xdict, pdict=None, asarray=False)  Direct access to a generator's Rhs function. | source code |

|  |  |
| --- | --- |
| Jacobian(self, t, xdict, pdict=None, asarray=False)  Direct access to a generator's Jacobian function (if defined). | source code |

|  |  |
| --- | --- |
| JacobianP(self, t, xdict, pdict=None, asarray=False)  Direct access to a generator's JacobianP function (if defined). | source code |

|  |  |
| --- | --- |
| MassMatrix(self, t, xdict, pdict=None, asarray=False)  Direct access to a generator's MassMatrix function (if defined). | source code |

|  |  |
| --- | --- |
| AuxVars(self, t, xdict, pdict=None, asarray=False)  Direct access to a generator's auxiliary variables definition (if defined). | source code |

|  |  |
| --- | --- |
| compute(self, trajname, \*\*kw)  Compute a non-hybrid trajectory. | source code |

|  |  |
| --- | --- |
| \_validateRegistry(self, obsvars, intvars)  Validate Model's modelInfo attribute. | source code |

|  |  |
| --- | --- |
| \_infostr(self, verbose=1)  Return string information about named sub-model (if given by dsName) at given verbosity level (default 0) | source code |

**Inherited from `Model`**:
`__call__`,
`__copy__`,
`__deepcopy__`,
`__delitem__`,
`__getitem__`,
`__len__`,
`__repr__`,
`__str__`,
`current_defining_args`,
`defaultVars`,
`forceIntVars`,
`forceObsVars`,
`getDSAlgPars`,
`getDSEventActive`,
`getDSEventTerm`,
`getEndPoint`,
`getEndTime`,
`getEventMappings`,
`getTrajEventStruct`,
`getTrajEventTimes`,
`getTrajEvents`,
`getTrajModelName`,
`getTrajTimeInterval`,
`getTrajTimePartitions`,
`has_exact_traj`,
`info`,
`query`,
`renameTraj`,
`resetEventTimes`,
`sample`,
`searchForNames`,
`searchForVars`,
`set`,
`setDSAlgPars`,
`setDSEventActive`,
`setDSEventBisect`,
`setDSEventDelay`,
`setDSEventDir`,
`setDSEventICs`,
`setDSEventInterval`,
`setDSEventPrecise`,
`setDSEventStartTime`,
`setDSEventTerm`,
`setDSEventTol`,
`setICs`,
`setPars`,
`showAuxFnSpec`,
`showAuxSpec`,
`showDSEventInfo`,
`showDef`,
`showEventSpec`,
`showSpec`,
`sub_models`

**Inherited from `Model`** (private):
`_delTraj`,
`_generateParamInfo`,
`_makeDefaultVarNames`,
`_prepareCompute`,
`_set_for_hybrid_DS`,
`_validateVarNames`

**Inherited from `object`**:
`__delattr__`,
`__getattribute__`,
`__hash__`,
`__new__`,
`__reduce__`,
`__reduce_ex__`,
`__setattr__`


|  |  |
| --- | --- |
| Class Variables | [hide private] |

**Inherited from `Model`** (private):
`_needKeys`,
`_optionalKeys`,
`_querykeys`,
`_setkeys`


|  |  |
| --- | --- |
| Properties | [hide private] |

**Inherited from `object`**:
`__class__`


|  |  |
| --- | --- |
| Method Details | [hide private] |

|  |  |
| --- | --- |
| \_\_init\_\_(self, \*a, \*\*kw)  *(Constructor)* | source code |

x.\_\_init\_\_(...) initializes x; see x.\_\_class\_\_.\_\_doc\_\_ for
signature

Overrides: object.\_\_init\_\_: *(inherited documentation)*

|  |  |
| --- | --- |
| cleanupMemory(self) | source code |

Clean up memory usage from past runs of a solver that is interfaced
through a dynamic link library. This will prevent the 'continue'
integration option from being accessible and will delete other data about
the last integration run.

|  |  |
| --- | --- |
| Rhs(self, t, xdict, pdict=None, asarray=False) | source code |

```
Direct access to a generator's Rhs function.
Parameters:

  t        time (can use 0 for an autonomous system)
  xdict    state dictionary or Point.
  pdict    parameter dictionary or Point
           (optional, default current parameters)
  asarray  (Bool, optional, default False) If true, will return an array
           in state name alphabetical order, else a Point
```

|  |  |
| --- | --- |
| Jacobian(self, t, xdict, pdict=None, asarray=False) | source code |

```
Direct access to a generator's Jacobian function (if defined).

Arguments:

  t        time (can use 0 for an autonomous system)
  xdict    state dictionary or Point.
  pdict    parameter dictionary or Point
           (optional, default current parameters)
  asarray  (Bool, optional, default False) If true, will return an array
           in state name alphabetical order, else a Point
```

|  |  |
| --- | --- |
| JacobianP(self, t, xdict, pdict=None, asarray=False) | source code |

```
Direct access to a generator's JacobianP function (if defined).

Arguments:

  t        time (can use 0 for an autonomous system)
  xdict    state dictionary or Point.
  pdict    parameter dictionary or Point
           (optional, default current parameters)
  asarray  (Bool, optional, default False) If true, will return an array
           in state name alphabetical order, else a Point
```

|  |  |
| --- | --- |
| MassMatrix(self, t, xdict, pdict=None, asarray=False) | source code |

```
Direct access to a generator's MassMatrix function (if defined).

Arguments:

  t        time (can use 0 for an autonomous system)
  xdict    state dictionary or Point.
  pdict    parameter dictionary or Point
           (optional, default current parameters)
  asarray  (Bool, optional, default False) If true, will return an array
           in state name alphabetical order, else a Point
```

|  |  |
| --- | --- |
| AuxVars(self, t, xdict, pdict=None, asarray=False) | source code |

```
Direct access to a generator's auxiliary variables
definition (if defined).

Arguments:

  t        time (can use 0 for an autonomous system)
  xdict    state dictionary or Point.
  pdict    parameter dictionary or Point
           (optional, default current parameters)
  asarray  (Bool, optional, default False) If true, will return an array
           in state name alphabetical order, else a Point
```

|  |  |
| --- | --- |
| compute(self, trajname, \*\*kw) | source code |

```
Compute a non-hybrid trajectory. Returns a Trajectory object.

Arguments (non-keyword):
  trajname   Name of trajectory to create (string)

Arguments (keyword only, all optional):
  force      (Bool, default False) - force overwrite of any trajectory
             stored in this object with the same name
  verboselevel  (int, default 0)
  ics        initial conditions dict or Point
  pars       parameters dict or Point
  tdata      time data (interval as sequence of 2 numeric values)
```

|  |  |
| --- | --- |
| \_infostr(self, verbose=1) | source code |

Return string information about named sub-model (if given by dsName)
at given verbosity level (default 0)

Overrides: Model.\_infostr: *(inherited documentation)*

  


| Home | Trees | Indices | Help | PyDSTool |
| --- | --- | --- | --- | --- |

|  |  |
| --- | --- |
| Generated by Epydoc 3.0.1 on Fri May 4 15:24:07 2012 | http://epydoc.sourceforge.net |
